# Evaluating Survival After Hospitalization Due to Immune-Related Adverse Events From Checkpoint Inhibitors

**DOI:** 10.1093/oncolo/oyad135

**Published:** 2023-06-19

**Authors:** Jordyn Silverstein, Francis Wright, Michelle Wang, Arabella Young, Daniel Kim, Kimberly De Dios, Sam Brondfield, Zoe Quandt

**Affiliations:** Department of Internal Medicine, University of California, San Francisco, San Francisco, CA, USA; Department of Internal Medicine, University of California, San Francisco, San Francisco, CA, USA; Bakar Computational Health Sciences Institute, University of California, San Francisco, San Francisco, CA, USA; Graduate Program in Pharmaceutical Sciences and Pharmacogenomics, University of California, San Francisco, San Francisco, CA, USA; Huntsman Cancer Institute, University of Utah Health Sciences Center, Salt Lake City, UT, USA; Department of Pathology, University of Utah School of Medicine, Salt Lake City, UT, USA; Department of Internal Medicine, University of California, San Francisco, San Francisco, CA, USA; Division of Endocrinology, Metabolism and Diabetes, Department of Medicine, University of California, San Francisco, San Francisco, CA, USA; Diabetes Center, University of California, San Francisco, San Francisco, CA, USA; Helen Diller Family Comprehensive Cancer Center, University of California, San Francisco, San Francisco, CA, USA; Department of Medicine, Division of Hematology and Oncology, University of California, San Francisco, San Francisco, CA, USA; Division of Endocrinology, Metabolism and Diabetes, Department of Medicine, University of California, San Francisco, San Francisco, CA, USA; Diabetes Center, University of California, San Francisco, San Francisco, CA, USA

**Keywords:** irAE, immunotherapy, immune checkpoint inhibitors, hospitalization, cancer

## Abstract

**Background:**

As immune checkpoint inhibitors (CPI) are increasingly approved for cancer treatment, hospitalizations related to severe immune-related adverse events (irAE) will increase. Here, we identify patients hospitalized due to irAEs and describe survival outcomes across irAE, CPI, and cancer type.

**Methods:**

We identified patients hospitalized at our institution from January 2012 to December 2020 due to irAEs. Survival was analyzed using Kaplan-Meier survival curves with log-rank tests.

**Results:**

Of 3137 patients treated with CPIs, 114 (3.6%) were hospitalized for irAEs, resulting in 124 hospitalizations. Gastrointestinal (GI)/hepatic, endocrine, and pulmonary irAEs were the most common causes of irAE-related hospitalization. After CPI initiation, the average time to hospitalization was 141 days. Median survival from hospital admission was 980 days. Patients hospitalized due to GI/hepatic and endocrine irAEs had longer median survival than patients with pulmonary irAEs (795 and 949 days vs. 83 days [*P* < .001]). Patients with melanoma and renal cell carcinoma had longer median survival than patients with lung cancer (2792 days and not reached vs. 159 days [*P* < .001]). There was longer median survival in the combination group compared to the PD-(L)1 group (1471 vs. 529 days [*P* = .04]).

**Conclusions:**

As CPI use increases, irAE-related hospitalizations will as well. These findings suggest that among patients hospitalized for irAEs, survival differs by irAE and cancer type, with worse survival for patients with irAE pneumonitis or lung cancer. This real-world data contributes to research pertaining to hospitalization due to severe irAEs, which may inform patient counseling and treatment decision-making.

## Teaser

Immune checkpoint inhibitor use is increasing, and immune-related adverse event (irAE)-related hospitalizations are also expected to increase. This article evaluates survival after hospitalization from an irAE. Findings suggest that among patients hospitalized for irAEs, survival differs by adverse event and cancer type.

**Implications for Practice**

Checkpoint inhibitor use is increasing and although rare, immune-related adverse event (irAE)-related hospitalizations will also increase. This is one of the first studies to evaluate survival after hospitalization from an irAE. These findings suggest that among patients hospitalized for irAEs, survival differs by irAE and cancer type, with worse survival for patients with irAE pneumonitis or lung cancer. This real-world data contributes to research pertaining to hospitalization due to severe irAEs, which may inform patient counseling and treatment decision-making.

## Introduction

Immune checkpoint inhibitors (CPI) are transforming the landscape of cancer care and are even approved for tumor-agnostic indications.^1-3^ These medications block the interaction of immune checkpoint proteins on tumor cells, thus allowing for immune activation^4^; they include inhibition of programmed cell death protein 1 (PD-1), programmed death-ligand 1 (PD-L1), cytotoxic T lymphocyte-associated 4 (CTLA-4), and most recently, lymphocyte activation gene-3 (LAG3).^5^

Although these treatments are overall better tolerated than conventional chemotherapies,^6^ the side-effect profile poses unique challenges. Side effects, called immune-related adverse events (irAEs), result from immune activation and can occur in almost any organ system.^7^ Rates of severe irAEs (Grade 3 or above) vary by treatment regimen, but up to 55% of patients treated with combination ipilimumab and nivolumab may develop a severe irAE.^8,9^

A subset of patients requires hospitalization for irAEs. A national study using insurance claims estimates 3.5% of patients initiating CPI therapy experience an irAE requiring hospitalization.^10^ Another study described 450 ­irAE-related hospitalizations; gastrointestinal (GI), pulmonary, hepatic, and endocrine irAEs were the most common.^11^ The irAE-specific mortality rate was 5.6% and highest for pulmonary and cardiac irAEs, higher than the overall reported incidence of fatal irAEs (0.3%-1.3%).^12^ Another study looked at 23 patients hospitalized for irAEs, of whom 3 (13%) died from the irAE.^13^ How the survival of patients hospitalized for irAEs might vary across multiple characteristics including CPI type, cancer type, and irAE remains poorly understood.

This study characterizes the spectrum of toxicities and survival of patients hospitalized for irAEs, as well as how survival varies across CPI type, cancer type, and irAE. Stratifying hospitalized patients in this way contributes to a more nuanced understanding of survival outcomes for patients hospitalized due to irAEs.

## Materials and Methods

This study was approved by the UCSF Human Research Protection Program [#17-22987].

### Patient Selection and Data Collection

**Figure 1.**
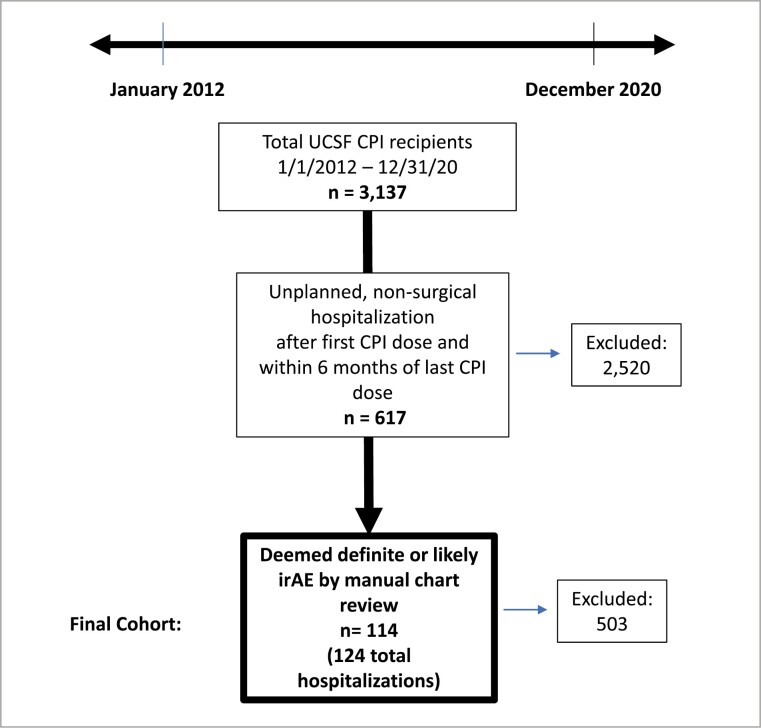
Inclusion criteria for the study cohort.

Inclusion criteria are summarized in Fig. 1. The study used computational extraction to identify patients with solid tumor malignancies who received CPIs from 1/1/2012 to 12/31/2020 and hospitalized any time between treatment initiation and 6 months after the last CPI dose. Hospitalizations for surgical procedures or planned chemotherapy were excluded. The remaining charts were manually reviewed by either a trained medical student (F.W., D.K.), resident (J.S.), or faculty member (Z.Q.) to include only definite or likely ­irAE-related hospitalizations. Ambiguous cases were discussed with a board-certified oncologist (S.B.). A confirmed irAE hospitalization was defined as consensus around the irAE diagnosis between the inpatient oncology team, the outpatient primary oncologist, and exclusion of alternative diagnoses. In cases where a biopsy was taken, the biopsies were reviewed to confirm the diagnosis. Cases that remained unclear after manual chart review and discussion were excluded (*n* = 36).

### Statistical Variables and Analysis

Relevant demographics, clinical history, and key admission characteristics were manually collected and stored using REDCap. Variables were summarized using means, medians, and interquartile ranges (IQRs) for continuous variables and proportions for categorical variables. Categorical and continuous variables were compared by CPI type using Fisher’s exact tests and Wilcoxon rank-sum tests, respectively. For ­post-hospitalization survival, follow-up was measured from the date of hospital admission to the date of death or last follow-up, with survival censored at the last follow-up, by Kaplan-Meier survival curves and log-rank tests. Survival was assessed for the overall cohort and stratified by CPI type, cancer type, and irAE type. Only subgroups that were greater than or equal to 10 were included. Observed ­post-hospitalization patient survival and 95% confidence intervals (CIs) were estimated at 1, 3, and 5 years. A sensitivity analysis using the date of CPI initiation to death or last follow-up was also completed. Statistical analyses were performed using Stata. Statistical significance was defined as *P*-value of ≤.05.

## Results

### Overall Demographic and Clinical Characteristics

**Figure 2.**
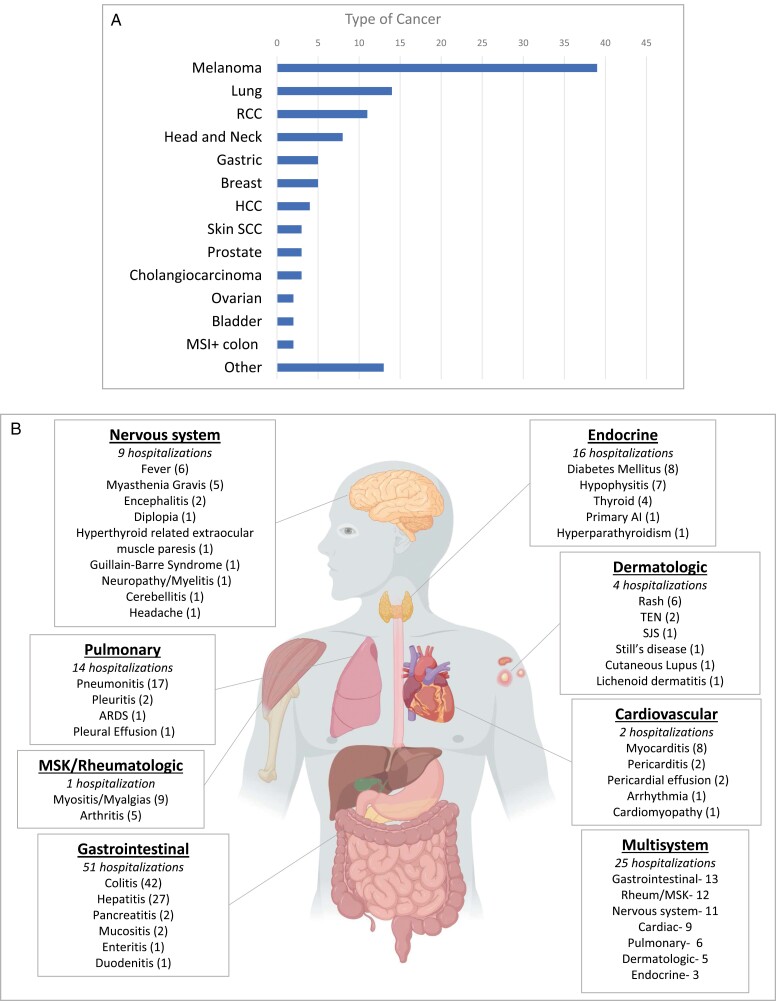
(**A**) Distribution of cancer type for patients hospitalized for immune-related adverse events (irAEs). (**B**) Distribution of Hospitalized irAEs. In parenthesis, the number per irAE describes the total number of hospitalizations combining hospitalizations for single-system irAE with multisystem irAEs. For example, for cardiovascular there were 4 hospitalizations for cardiac irAE alone and 10 hospitalizations with cardiac irAEs combined with other system irAEs. A hospitalization within 1 system could have multiple specific irAEs within that system in 1 hospitalization. Number of hospitalizations in italics describe irAE hospitalizations with only that system.

Of 3137 patients treated with CPIs from 2012 to 2020, 114 were hospitalized for confirmed irAEs (cumulative incidence 3.6%) resulting in 124 total hospitalizations (Table 1). Of the 114 patients hospitalized for irAEs, 34.2% [39] had melanoma, 12.2% [14] had lung cancer, 9.6% [11] had renal cell carcinoma (RCC), 7.0% [8] had head and neck cancer, and the rest were distributed across other cancers (Fig. 2A). The average age was 61.5 (range: 22-91). The majority of patients were Caucasian (74.5%), had Eastern Cooperative Oncology group (ECOG) performance status 0 or 1 prior to hospitalization (88.1%), and did not have an autoimmune condition prior to the initiation of therapy (85.9%). Autoimmune conditions by year are reported in Supplementary Fig. 2A. Three patients had undergone transplants (kidney, pancreas and kidney, and stem cell).

**Table 1.** Baseline demographic and clinical information by CPI type.

	Overall cohort	PD-(L)1 monotherapy	Combination therapy (PD-[L]1/CTLA-4)	CTLA-4 monotherapy	*P*-value^*^
	(*n* = 114)	(*n* = 69)	(*n* = 39)	(*n* = 6)	
Age (years) (range)	61.5 (22-91)	62.8 (22-91)	58.9 (24-81)	62.2 (49-73)	.25
BMI (range)	26.4 (15.4-45.7)	25.5 (15.4-45.7)	27.8 (20.7-37.6)	28.6 (20.0-39.3)	.08
Gender (%)					
Male	66 (57.8)	34 (49.3)	29 (74.4)	3 (50.0)	
Female	47 (41.2)	34 (49.3)	10 (25.6)	3 (50.0)	
Nonbinary	1 (0.9)	1 (1.4)	0 (0)	0 (0)	.06
Race/ethnicity (%)		45 (65.2)	35 (89.7)		
Caucasian	85 (74.6))	9 (13.0)	3 (7.7)	5 (83.3)	
Hispanic	13 (11.4)	3 (4.3)	0 (0)	1 (16.6)	
African American	3 (2.6)	11 (15.9)	1 (2.6)	0 (0)	
Asian	12 (10.5)	2 (2.9)	0 (0)	0 (0)	
American Indian/	4 (3.5)			0 (0)	
Alaska Native		1 (1.4)	0(0)		
Unknown	1(0.8)			0(0)	.01^**^
ECOG at first CPI					
0-1	101 (88.6)	59 (85.5)	36 (92.3)	6 (100.0)	
**≥**2 or unknown	13 (11.4)	10 (14.5)	3 (7.7)	0 (0)	.35
Autoimmune condition					
Present, on immunomodulatory therapy	4 (3.5)	3 (4.3)	1 (2.6)	0 (0)	
Present, not on immunomodulatory therapy	12 (10.5)	9 (13.0)	3 (7.7)	0 (0)	
Absent	98 (85.9)	57 (82.6)	35 (89.7)	6 (100.0)	.84
Charlson comorbidity score (%)					
≤4	11 (9.6)	7 (10.1)	4 (10.2)	0 (0)	
05 August	59 (51.7)	35 (50.7)	20 (51.3)	4 (66.6)	
≥9	44 (38.6)	27 (39.1)	15 (38.4)	2 (33.3)	1
Number of irAE hospitalizations (%)					
1	95 (83.3)	56 (81.1)	35 (89.7)	4 (66.7)	
2	15 (13.1)	9 (13.0)	4 (10.3)	2 (33.3)	
3	3 (2.6)	3 (4.3)	0 (0)	0 (0)	
4	0 (0)	0 (0)	0 (0)	0 (0)	
5	1 (0.9)	1 (1.4)	0 (0)	0 (0)	.22^***^
Time to hospitalization					
Mean, days (range)	140.8 (4-1329)	175.1 (8-1329)	85.0 (4-351)	66.0 (20-118)	
Median, days	76.5	98	69	67	.07
Type of cancer (%)					
Melanoma	39 (34.2)	11 (15.9)	22 (56.4)	6 (100.0)	
Lung	14 (12.3)	11 (15.9)	3 (7.7)	0 (0)	
Head and neck	8 (7.0)	6 (8.5)	2 (5.1)	0 (0)	
RCC	11 (9.6)	6 (7.0)	5 (12.8)	0 (0)	
Bladder	2 (1.8)	2 (2.8)	0 (0)	0 (0)	
Gastric	5 (4.4)	5 (8.5)	0 (0)	0 (0)	
MSI + colon	2 (1.8)	0 (0)	2 (5.1)	0 (0)	
Breast	5 (4.4)	5 (7.0)	0 (0)	0 (0)	
Skin SCC	3 (2.6)	3 (4.2)	0 (0)	0 (0)	
HCC	4 (3.5)	4 (5.6)	0 (0)	0 (0)	
Ovarian	2 (1.8)	2 (2.8)	0 (0)	0 (0)	
Prostate	3 (2.6)	2 (2.8)	1 (2.6)	0 (0)	
Cholangiocarcinoma	3 (2.6)	3 (4.2)	0 (0)	0 (0)	
Other	13 (11.4)	10 (14.1)	4 (10.3)	0 (0)	<.001
CPI type					
Combination CPI	38 (3.3)	0 (0)	38 (97.4)	0 (0)	
CPI Monotherapy	55 (48.2)	49 (71.0)	0 (0)	6 (100.0)	
CPI plus Chemo	12 (0.5)	11 (15.9)	1 (2.6)	0 (0)	
CPI plus other immunomodulator	9 (7.9)	9 (13.0)	0 (0)	0 (0)	<.001
CPI drug					
Ipilimumab	6 (5.2)	0 (0)	0 (0)	6 (100.0)	
Nivolumab	23 (20.2)	23 (33.3)	0 (0)	0 (0)	
Ipi/Nivo	35 (30.7)	0 (0)	35 (89.7)	0 (0)	
Pembrolizumab	39 (34.2)	39 (56.5)	0 (0)	0 (0)	
Durvalumab	2 (1.7)	2 (2.9)	0 (0)	0 (0)	
Atezolizumab	3 (2.6)	3 (4.3)	0 (0)	0 (0)	
Cemiplimab	2 (1.7)	2 (2.9)	0 (0)	0 (0)	
Ipi/Pembro	3 (2.6)	0 (0)	3 (7.7)	0 (0)	
Tremelimumab/Durva	1 (0.9)	0 (0)	1 (2.6)	0 (0)	<.001
Part of a clinical trial?					
Yes	29 (25.4)	23 (33.3)	5 (12.8)	1 (16.6)	
No	85 (74.5)	46 (66.6)	34 (87.2)	5 (83.3)	.05
Metastatic or unresectable at CPI initiation					
Yes	103 (90.3)	62 (89.8)	35 (89.7)	6 (100.0)	
No	11 (9.6)	7 (10.1)	4 (10.3)	0 (0)	1
Stage (%)					
Stage I	3 (2.6)	2 (2.9)	1 (2.6)	0 (0)	
Stage II	6 (5.2)	5 (7.2)	1 (2.6)	0 (0)	
Stage III	14 (12.2)	9 (13.0)	5 (12.8)	0 (0)	
Stage IV	90 (78.9)	53 (76.8)	31 (79.5)	6 (100.0)	
Unknown	1 (0.9)	0 (0)	1 (2.6)	0 (0)	.85
Prior lines of therapy					
None	49 (42.9)	24 (34.7)	22 (56.4)	3 (50.0)	
1	37 (32.5)	23 (33.3)	11 (28.2)	3 (50.0)	
2	14 (12.2)	12 (17.3)	2 (5.1)	0 (0)	
3 or more	14 (12.2)	10 (14.4)	4 (10.3)	0 (0)	.58
Prior CPI therapy?					
Yes	15 (13.1)	8 (11.6)	7 (17.9)	0 (0)	
No	99 (86.8)	61 (88.4)	32 (82.1)	6 (100.0)	.35

^*^The *P*-value compares the difference between the CPI treatment groups

^**^Comparing Caucasian to all other races/ethnicities combined, some patients considered themselves both Hispanic and another race.

^***^ *P*-value is categorical comparing 1 hospitalization versus more than 1 hospitalization.

Abbreviations: BMI, body mass index; CPI, checkpoint inhibitor; Durva, durvalumab; HCC, hepatocellular carcinoma; Ipi, ipilimumab; irAE, immune-related adverse event; Nivo, nivolumab; Pembro, pembrolizumab; RCC, renal cell carninoma; SCC, squamous cell carcinoma.

The cohort consisted almost entirely of patients with metastatic or unresectable disease (90.3%) at the time of CPI initiation. CPI was the first line of therapy for 42.9% while 12.2% had undergone 3 or more lines of therapy. The types of CPI included pembrolizumab monotherapy (34.2%), combination ipilimumab/nivolumab (30.7%), nivolumab monotherapy (20.2%), and other monotherapies (ipilimumab [6], durvalumab [2], atezolizumab [3], cemiplimab [2]) or combination regimens (ipilimumab/pembrolizumab [3] or tremelimumab/durvalumab [1]).

### Demographic and Clinical Characteristics by CPI Type

When grouped by CPI type, the groups varied significantly only by type of cancer and race (Table 1). In the PD-(L)1 group (*n* = 69), 15.9% [11] had melanoma, 15.9% [11] lung cancer, 8.7% [6] RCC, 8.7% [6] head and neck cancer, 7.2% [5] gastric cancer, 7.2% [5] breast, and 4.3% [3] cutaneous squamous cell carcinoma (SCC). In the combination group (PD-[L]1/CTLA-4; *n* = 39), melanoma (56.4% [22]) and RCC (12.8% [5]) were the most common cancers. While the cohort was predominantly Caucasian, the PD-(L)1 monotherapy group had the most other races/ethnicities (34.8%). When comparing the combination group to the PD-(L)1 group, there were significantly more males in the combination group (74.4%) than in the PD-(L)1 group (49.3%; *P* = .04).

### irAE Characteristics

The average number of irAE-related hospitalizations per patient was 1.09 (ranging from 1 to 3). A summary of the different irAEs by system can be found in Fig. 2B. Among 124 hospitalizations, 41.2% [51] were due to GI irAEs including hepatitis, 12.9% [16] endocrine, 11.2% [14] pulmonary, 7.2% [9] neurologic, 3.2% [4] dermatologic, 1.6% [2] cardiovascular, and 0.8% [1] rheumatologic/ musculoskeletal. Multisystem irAEs accounted for 20.2% [25] of irAE-related hospitalizations. The mean time to hospitalization was 141 days, and 41.1% of the hospitalizations occurred less than 60 days after CPI initiation. The CTLA-4 group had the shortest median time to hospitalization at 66.0 days (IQR: 20-118), followed by the combination group at 85.0 days (4-351) and the PD-(L)1 group at 175.1 days (8-1329; *P* = .07). The most common specific irAEs were colitis [42], pneumonitis [17], hepatitis [27], myocarditis [8], myositis/myalgias [8], diabetes mellitus [8], and hypophysitis [5]. IrAE treatments included steroids (84.7%), infliximab (12.9%), intravenous immunoglobulin (10.4%), and mycophenolate (2.4%).

### Gastrointestinal irAEs

GI irAEs accounted for 41.1% [51] of hospitalizations, 46 unique patients. The most common GI irAEs were colitis [31], hepatitis [12], combined colitis/hepatitis [4], colitis/duodenitis [1], colitis/enteritis [1], and pancreatitis [2]. For colitis, 21 out of the 37 hospitalizations underwent colonoscopies with biopsy were done to aid in diagnosis. The median time to hospitalization from CPI initiation was 84 days (range 20-1212). Of the 51 hospitalizations, 19 [37.2%] patients had melanoma, 5 [9.8%] lung, 6 [11.8%] RCC, and 4 [7.8%] head and neck SCC. 25 [49.0%] hospitalizations were from PD-1/PD-L1 monotherapy, 21 [41.1%] were from combination PD-1/CTLA-4 and 5 [9.8%] were from CTLA-4 monotherapy.

The median survival after GI irAE hospitalization was 795 days. Hospitalizations from colitis alone had a median survival of 686 days and hepatitis median survival of 540 days. The 1-year overall survival (OS) for all GI irAEs combined was 64.2% (95% CI, 49.3-75.7), 3-year OS was 48.6% (33.3-62.4), and 5-year OS was 38.1% (22.7-53.4). For colitis and hepatitis, the 1-year OS was 60.5% (40.9-75.4) and 54.6% (22.9-78.0), respectively. Steroids were used as treatment in 94.1% [48] and infliximab was used in 29.4% [15] of GI irAE hospitalizations. All the infliximab was used to treat colitis.

### Endocrine irAEs

Endocrine irAEs, accounted for 16 out of 124 hospitalizations, 16 unique patients. Endocrine irAEs included diabetes [8], hypophysitis [5], and thyroid dysfunction [2]. The median time to hospitalization was 111 days (range: 21-285). Of these 16 patients, 5 had melanoma and 2 had RCC. Ten of the 16 were treated with PD-1/PD-L1 inhibitors and 6 with combination PD-1/CTLA-4. The median survival was 949.7 days. Post-hospitalization 1-year survival was 87.5% (95% CI, 58.6-96.7) and 3-year survival was 46.3% (18.3-70.5). Four of the 16 hospitalizations (25.0%) with endocrine irAEs were treated with steroids beyond physiologic dosing.

### Pulmonary irAEs

Pulmonary irAEs accounted for 14 of the 126 hospitalizations, 14 unique patients. Pulmonary irAEs consisted of pneumonitis [13] and acute respiratory distress syndrome [1]. The median time to hospitalization was 75 days (range: 5-163, mean 76.2). Of these 14 patients, 4 [28.6%] had lung cancer and 2 [14.3%] had melanoma. Twelve out of the 14 [85.7%] hospitalizations were from PD-1/PD-L1 monotherapy. Of the 14 patients, 7 [50.0%] were either current or prior smokers and 5 [35.7%] had Charlson comorbidity scores of ≥9. However, there was no statistical difference in tobacco use (*P = .*53) or Charlson comorbidity (*P = .*49) between those who had a pulmonary irAE hospitalization and the rest of the irAE hospitalizations. Median survival after a pulmonary irAE was 82.5 days and the 1-year post-hospitalization survival was 28.6% (95% CI, 8.8-52.3). All 14 pulmonary irAE hospitalizations included steroids as treatment.

### Multisystem irAEs

There were 25 hospitalizations with multisystem irAEs in 24 unique patients. Among these patients, the mean number of irAEs was 2.5 (range: 2-6). The median time to hospitalization was 45.5 days (range: 15-1329, mean 154.6). The most common CPI type was PD-(L)1 monotherapy (72.0%). Of the 60 irAEs included in the multisystem group, 21.7% [13] were GI, 21.7% [12] were rheumatologic/musculoskeletal, 16.7% [10] were neurological, 15.0% [9] were cardiac, 10.0% [6] were pulmonary and the rest were dermatologic [5], endocrine [3], fatigue [1], and hematologic [1]. The most common combinations were neurological and rheumatologic/musculoskeletal in 4 patients, cardiac and pulmonary in 3 patients, and cardiac, rheumatologic/MSK, and GI in 3 patients. The most common specific irAEs within this multisystem group were hepatitis [11], myositis [9], myocarditis [7], colitis [5], and fever [5]. The median survival was 649.7 days, 1-year post-hospitalization survival was 58.9% (95% CI, 37.1-75.5) and 3-year survival was 49.1% (24.3-69.9).

### Overall Survival

**Figure 3.**
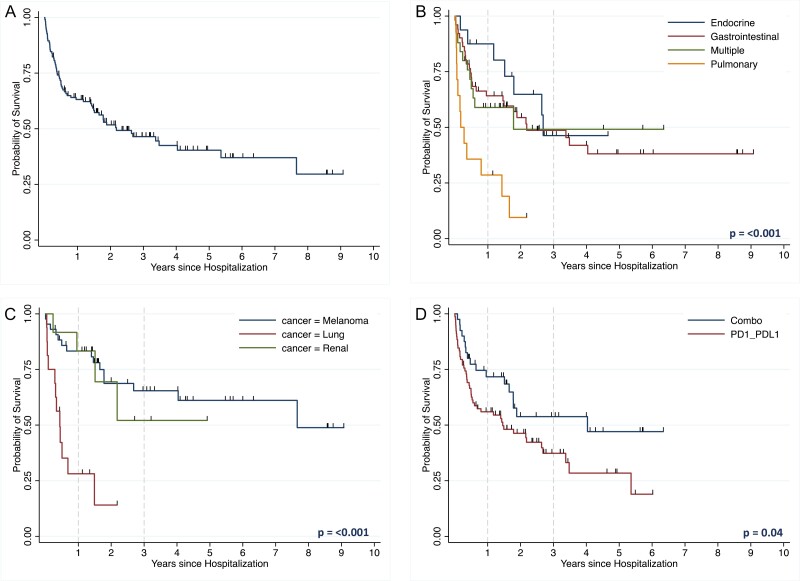
(**A**) Overall survival from time of hospitalization of the entire cohort. (**B**) Overall survival of immune-related adverse event (irAE)-related hospitalizations by irAE type. (**C**) Overall survival for irAE-related hospitalizations by cancer type. (**D**) Overall survival of patients hospitalized for irAE by checkpoint inhibitor type. A sensitivity analysis was performed with the same models but excluded patients with non-metastatic disease which had similar results to the overall cohort (Supplementary Figs. 4A–4D).

Across all patients hospitalized for irAEs, the median survival following hospitalization was 980 days. The 1-year overall survival was 63.2% (95% CI, 53.9-71.0), 3-year OS 46.5% (36.5-55.8) and 5-year OS 40.4% (29.8-50.7; Fig. 3A). At the time of chart review, the patients in 65 out of 124 hospitalizations had died. In 4.0% of hospitalizations [5], the patient died within 14 days of hospital admission. For 8.1% [10] of hospitalizations, the patient died within 30 days of admission.

### Post-hospitalization Survival by irAE Type

Patients with GI, endocrine, and multiple irAEs had significantly longer median survival (796 days, median not reached [NR], and 646 days, respectively), than patients with pulmonary irAEs (62 days; *P* = .003). The 1-year OS for GI, endocrine, multiple, and pulmonary irAEs was 64.2% (95% CI, 49.3-75.7), 87.5% (58.6-96.7), 58.9% (37.1-75.5), and 28.6% (8.8-52.4), respectively (Fig. 3B). There were significant differences in post-hospitalization survival between GI and pulmonary irAEs (*P* = .001), endocrine and pulmonary (*P* = .001), and multisystem and pulmonary (*P* = .04). Type of irAE treatment was not associated with differences in post-hospitalization survival for this group (Supplementary Figs. 3A–3D).

### Post-hospitalization Survival by Cancer Type

Cancers that affected 10 or more patients in the cohort were compared. Patients with melanoma and RCC had longer median survival after hospitalization compared to patients with lung cancer (2792 days and NR vs. 159 days, *P* < .001; Fig. 3C). Out of the patients with lung cancer (16), 37.5% (6) had pulmonary irAEs, and 31.3% (5) had GI irAEs, and only 5.9% (1) had an endocrine irAE. The 1-year post-hospitalization survival for patients with melanoma, RCC, and lung cancer was 83.3% (95% CI, 68.1-91.7), 83.3% (48.2-95.5), and 28.1% (8.9-51.4), respectively. The 1-year post-CPI initiation survival for patients with melanoma, RCC, and lung cancer was 88.4% (74.3-94.9), 91.7% (53.9-98.8), and 31.3% (11.4-53.7). Additionally, a subset analysis looked at the post-hospitalization survival by both cancer type and irAE. Patients with melanoma had similar trends to the overall cohort (see Supplementary Figs. 1A–1C).

### Post-hospitalization Survival by CPI Type

There was longer survival in the combination group (median 1471 days, *P* = .04) compared to the PD-(L)1 group (median 529 days; Fig. 3D). The post-hospitalization overall survival for the PD-(L)1 group and combination group was 56.0% (44.2-66.2) versus 71.7% (54.7-83.3) at 1 year, 37.3% (25.2-49.4) versus 53.7% (35.3-69.2) at 3 years, and 28.4.4% (15.3-43.0) versus 47.1% (27.1-64.8) at 5 years. Median overall survival was NR for the CTLA-4 monotherapy group. For patients with melanoma, CPI type was not associated with post-hospitalization survival (*P* = .29; Supplementary Fig. 1D).

## Discussion

This study describes the experience at UCSF with ­irAE-related hospitalizations. The frequency and distribution of irAEs across organ systems are consistent with prior studies ­reporting irAE-related hospitalizations.^11,13^ The most common types of irAEs that cause hospitalization were GI/hepatic, ­endocrine, and pulmonary, which are distinct from the most common irAEs of any grade which are pruritis, rash, and diarrhea not defined as colitis.^14^ This study adds nuance to the characterization of irAEs leading to hospitalization, reporting outcomes across cancer and CPI type in addition to irAE type.

This study characterized the type of patients hospitalized from irAEs at UCSF. In this study, 3.6% of patients treated with a CPI at UCSF were hospitalized from irAEs, which is consistent with a study that used national insurance claims.^10^ This number could underestimate the true percentage of patients hospitalized after irAEs since it only includes hospitalizations at UCSF. The catchment area of UCSF is broad, including 25 counties; therefore, it is possible patients could present to local hospitals for irAE treatment and not get transferred to UCSF, which would not be included in this study. Additionally, UCSF has created an urgent care for cancer center which promptly identifies and treats irAEs possibly preventing hospitalizations. The average age of patients in the cohort (62 years old) was similar to a population-level study of irAE-related hospitalizations.^10^ Most patients had metastatic or unresectable melanoma, lung cancer, or RCC at CPI initiation, which are most frequently treated with CPI therapy.^2^ While the majority of patients did not have a prior autoimmune disorder, 13.7% did, which is larger than was reported in a previous study on irAE-related hospitalizations (4.0%).^11^ History of an autoimmune condition has been shown to increase the risk of irAEs and hospitalization from irAEs.^15,16^ Additionally, 20% of hospitalizations had multiple irAEs. Therefore, if a patient has one irAE, it is important to evaluate for others.

This study assessed post-hospitalization survival after an irAE-related hospitalization stratified by CPI, cancer, and irAE type. The median survival following hospitalization was nearly 3 years, and 1-year post-hospitalization survival was 63%. Survival after hospitalization from irAEs differed by type of cancer type. In this study, patients with lung cancer who experienced an irAE hospitalization had worse OS. While this is likely due to the fact that lung cancer in general has worse OS,^17^ the survival could be lower due to the irAE itself in lung cancer patients. The 1-year survival in this study for patients with lung cancer and any irAE hospitalization was 30%, compared to a prior large study of patients with all stages of lung cancer who initiated CPI therapy had a 1-year survival of 43%.^18^ This is in contrast to patients with melanoma and RCC, who despite having an irAE hospitalization had similar overall survival (1-year post-hospitalization survival: 80%) to studies of patients on CPIs without hospitalization, which ranged from 65% to 80%.^19,20^ Many studies suggest that an irAE from a CPI can predict improved survival, as this could indicate response to therapy in lung cancer, RCC, and melanoma.^17,21-28^ Therefore, even if the irAE leads to hospitalization, future studies with a proper control group and sample size that accounts for important clinical factors such as cancer type, are needed to determine if irAE hospitalization could be predictive of overall survival outcomes.

In this study, post-hospitalization survival not only differed by cancer type, but also by irAE type. Patients hospitalized for pulmonary irAEs (33%) were shown to have worse post-hospitalization survival than those with GI (64%), endocrine (87%), or multisystem irAEs (57%). Although limited data exist describing survival following hospitalization from CPI-induced pneumonitis, retrospective studies of patients with lung cancer with severe CPI-induced pneumonitis report a 2.7-fold increased risk of death, and an OS of 3.0 months, which is similar to the current study.^29-31^ Pneumonitis likely confers a poor prognosis due to the nature of the irAE itself and may carry particular risks in individuals with lung cancer, smoking history, chronic obstructive pulmonary disease, or prior radiation to the chest.^30,32-34^ Patients with lung cancer have the highest incidence of pneumonitis, so it is likely that the inferior survival for CPI-induced pneumonitis and lung cancer overlap (40% of hospitalizations for pneumonitis had lung cancer in this study).^35^ In contrast, GI and endocrine irAEs, including grades ≥3, have been shown to be a predictor of better overall survival compared to patients treated with CPIs without irAEs.^36-42^ The mechanism by which some irAEs are associated with improved survival is hypothesized to be increased immunological activation leading to improved antitumor immunity.^7^

In this cohort, irAE-related hospitalizations occurred earlier after CPI initiation with regimens including CTLA-4 inhibitors and with GI or pulmonary irAEs, and later with PD-(L)1 monotherapy regimens and with endocrine irAEs. The time to hospitalization after CPI initiation for the overall cohort averaged 138 days, similar to a population-level study of severe irAEs (148 days).^10^ Although not statistically significant, there was a trend toward earlier irAE hospitalization with CTLA-4 monotherapy (66 days) or combination therapy CTLA-4/PD-(L)1 (85 days) compared to PD-(L)1 monotherapy (170 days). Other studies have shown that CTLA-4 therapy is correlated with shorter time to severe irAEs.^43^ Pulmonary (mainly pneumonitis) and GI (colitis and hepatitis) irAEs had a shorter median time to hospitalization at 77 and 84 days respectively, while endocrine irAEs had a longer time to hospitalization at 111 days. Prior studies have shown that CPI-induced pneumonitis had a median onset of 79 days,^44^ CPI colitis around 6-7 weeks, and CPI hepatitis around 6-14 weeks.^43,45^ The shorter onset of all-grade colitis in prior studies compared to this study of only high-grade colitis could reflect earlier lower-grade colitis preceding hospitalization and thus an opportunity to prevent hospitalization with early detection. Endocrine irAEs have been shown to occur later and take longer to resolve at up to 28 weeks after CPI initation.^43^ In this study, there was a large range of time to hospitalization for GI irAEs, with 1 patient experiencing a GI irAE resulting in hospitalization more than 3 years after CPI initiation. This suggests that severe irAEs can occur outside of the expected time window and thus clinicians should keep the possibility of an irAE in mind for all patients who have received CPI therapy. These patterns may help clinicians better recognize the expected time window for severe irAE onset, guiding earlier detection and prevention of hospitalization.

There are several limitations of the present study. Although this cohort was drawn from many years of patients treated at UCSF, the number of patients hospitalized for irAEs was small (*n* = 114), limiting the power to detect differences between subgroups and specifically by cancer type. Thus, only groups with greater than 10 patients were included in the subgroup analysis. Additionally, the findings of this single-institution study performed at an academic medical center may have limited generalizability to other practice settings. Since this study did not have a control group, further studies are needed to understand the difference in survival outcomes between patients who are treated with CPI therapy without a hospitalization compared to the patients in this study who are hospitalized for irAEs. Additionally, since there were only a small number of patients treated with CTLA-4 monotherapy, comparison by CPI type was limited. Lastly, this study cannot draw conclusions related to the cause of death because the study did not include the cause of death as a variable during chart review and data extraction.

## Conclusions

As CPI therapy is used more frequently, hospitalizations from irAEs will continue to increase. This study’s findings suggest that among patients hospitalized due to irAEs, survival may differ by irAE and cancer type, with particularly short survival for patients with lung cancer and/or irAE pneumonitis. This real-world data can contribute to clinical models that assess the outcomes of hospitalization and the risk of death due to severe irAEs, which may inform patient counseling and treatment decision-making.

## Supplementary Material

oyad135_suppl_Supplementary_Figures

## Data Availability

The data underlying this article will be shared on reasonable request to the corresponding author.

## Previous Presentation

Preliminary data were submitted and accepted as a poster presentation at the 2022 ASCO Annual Meeting.
