# Supplementary material for: Evaluating Survival After Hospitalization Due to Immune-Related Adverse Events From Checkpoint Inhibitors
Source: Oncologist. 2023 Jun 19;28(10):e950–9. doi: 10.1093/oncolo/oyad135 (PMC10546826; doi:10.1093/oncolo/oyad135)
Supplement: oyad135_suppl_Supplementary_Figures [file oyad135_suppl_supplementary_figures.pptx]

## Slide 1
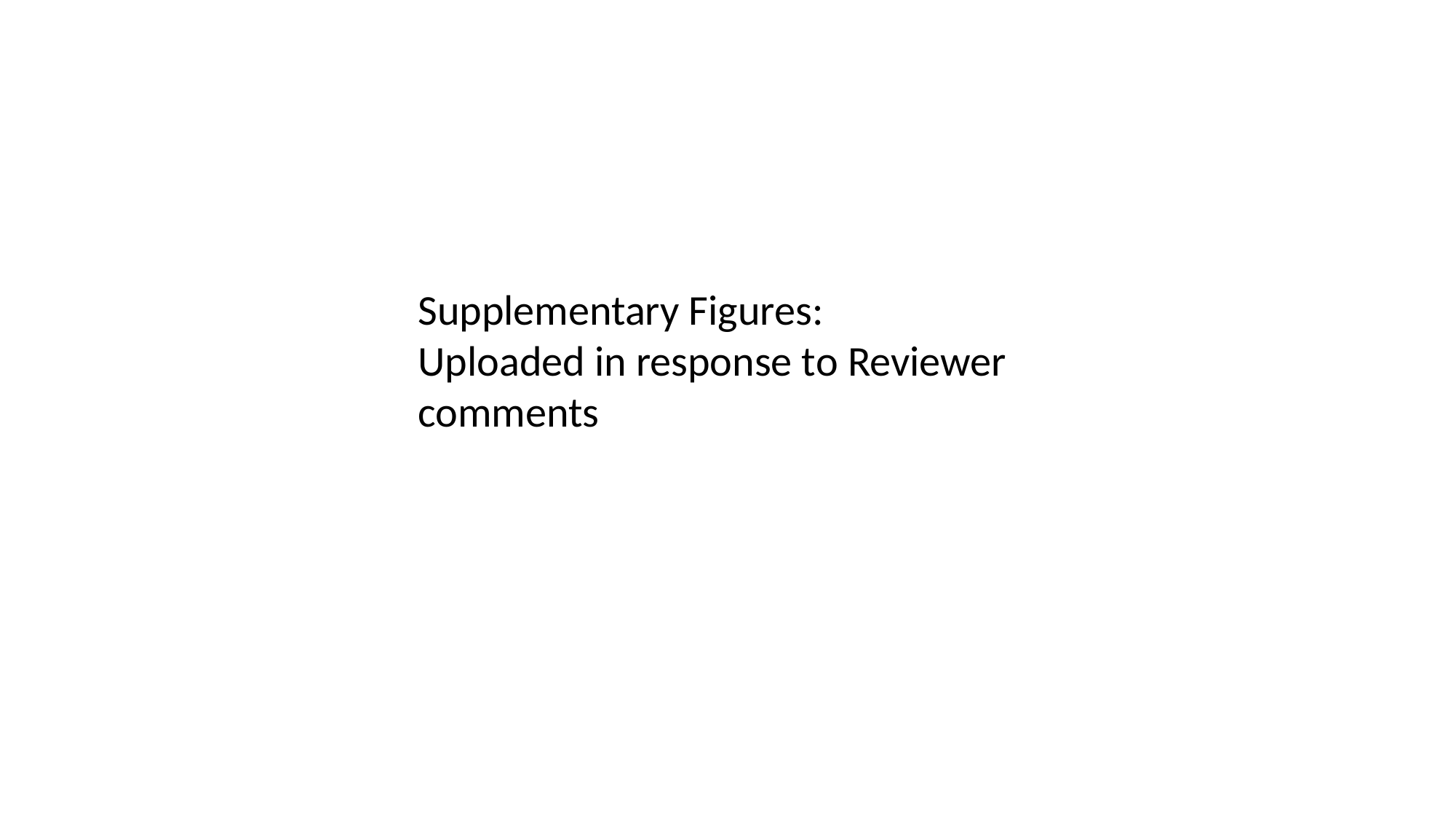

Supplementary Figures:
Uploaded in response to Reviewer comments

## Slide 2
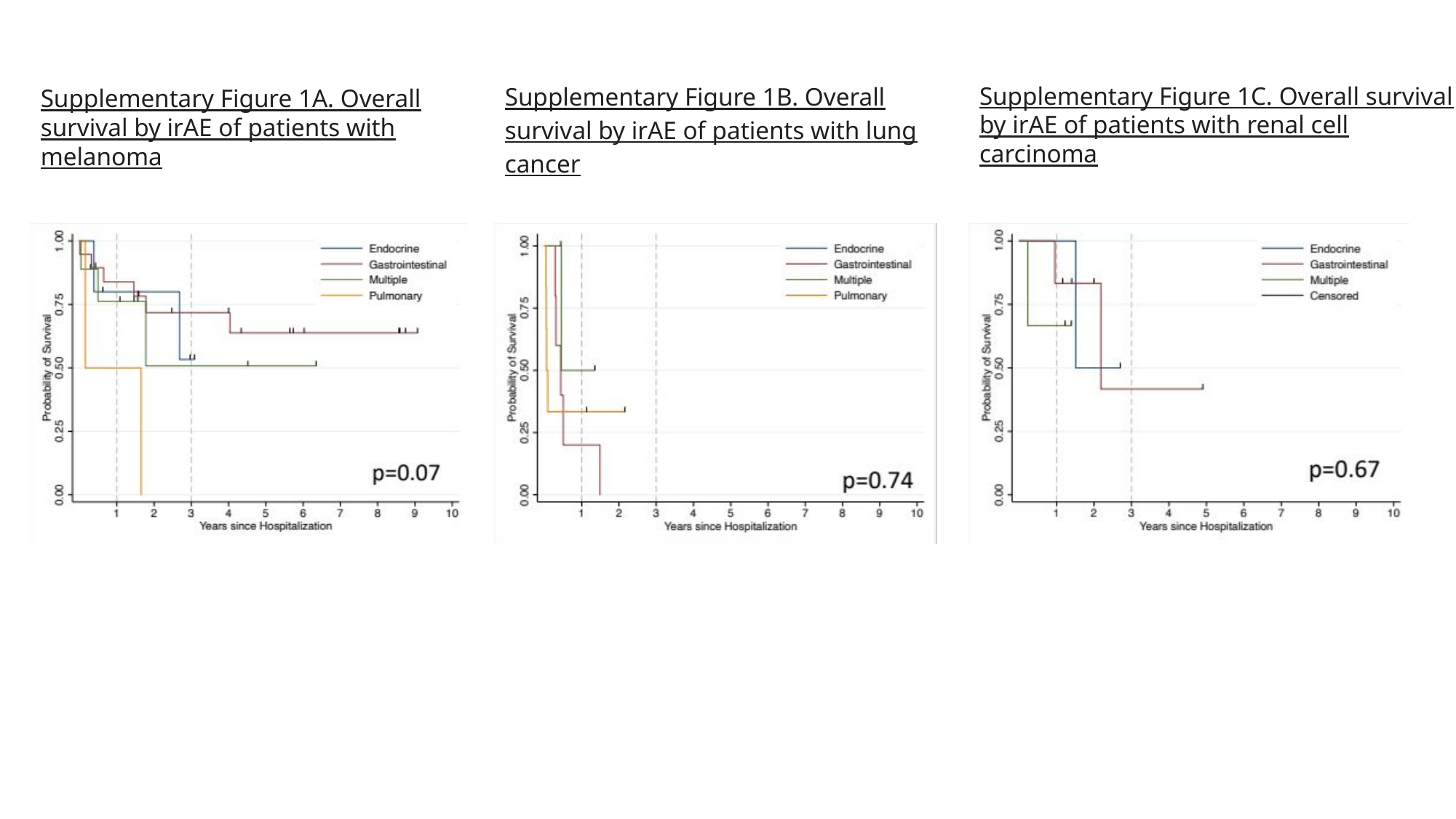

Supplementary Figure 1B. Overall survival by irAE of patients with lung cancer
Supplementary Figure 1C. Overall survival by irAE of patients with renal cell carcinoma
Supplementary Figure 1A. Overall survival by irAE of patients with melanoma

## Slide 3
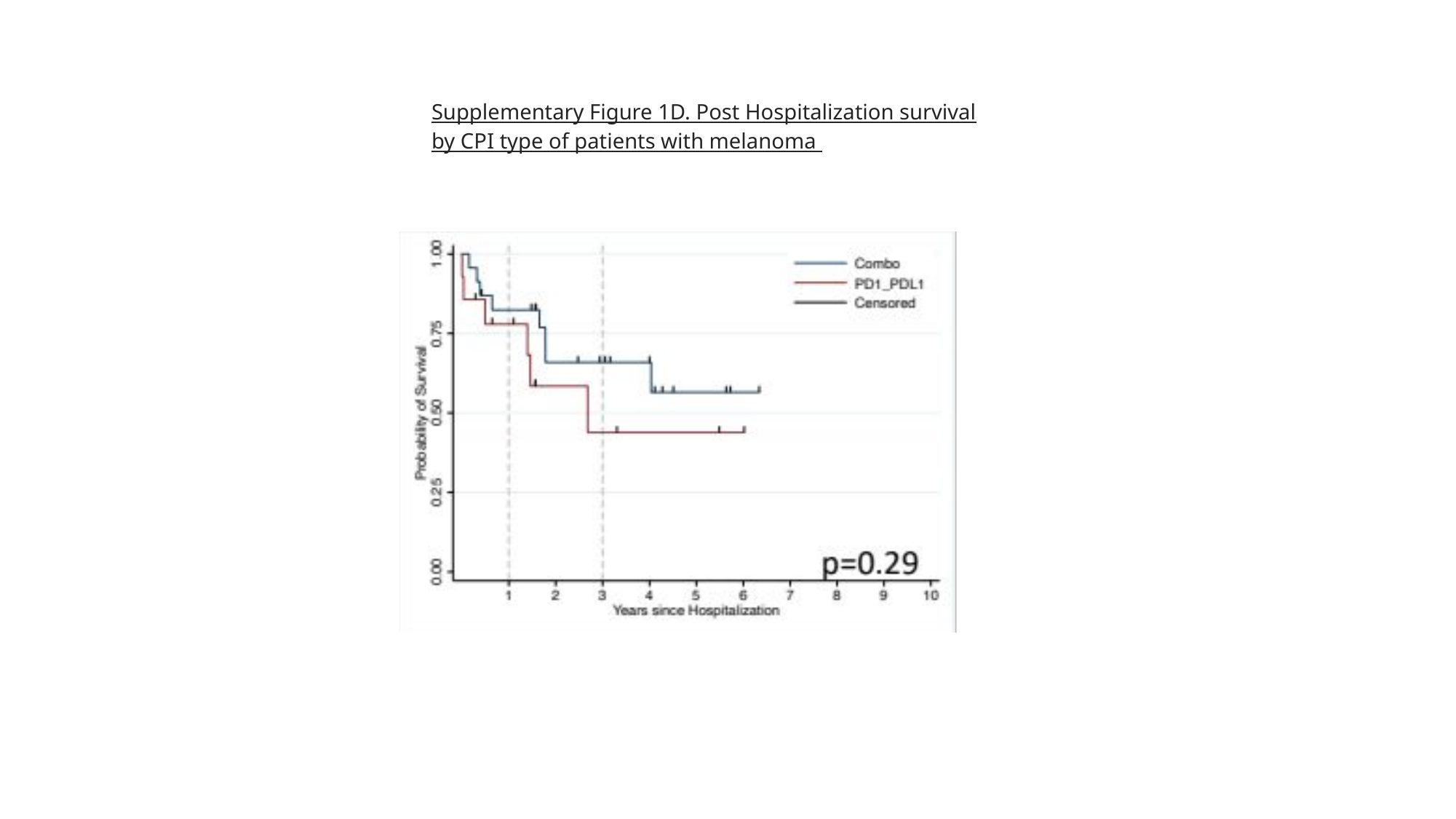

Supplementary Figure 1D. Post Hospitalization survival by CPI type of patients with melanoma

## Slide 4
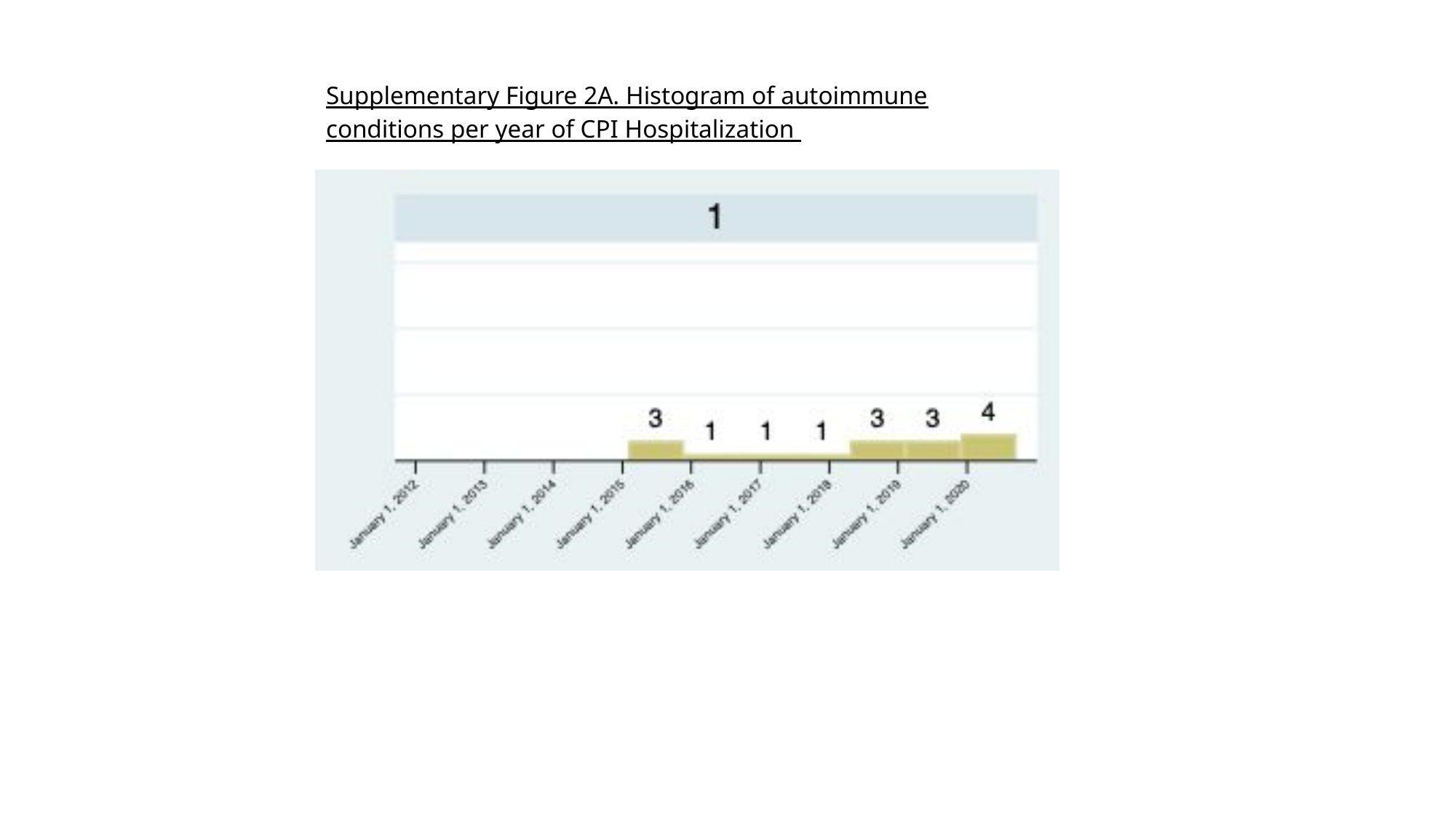

Supplementary Figure 2A. Histogram of autoimmune conditions per year of CPI Hospitalization

## Slide 5
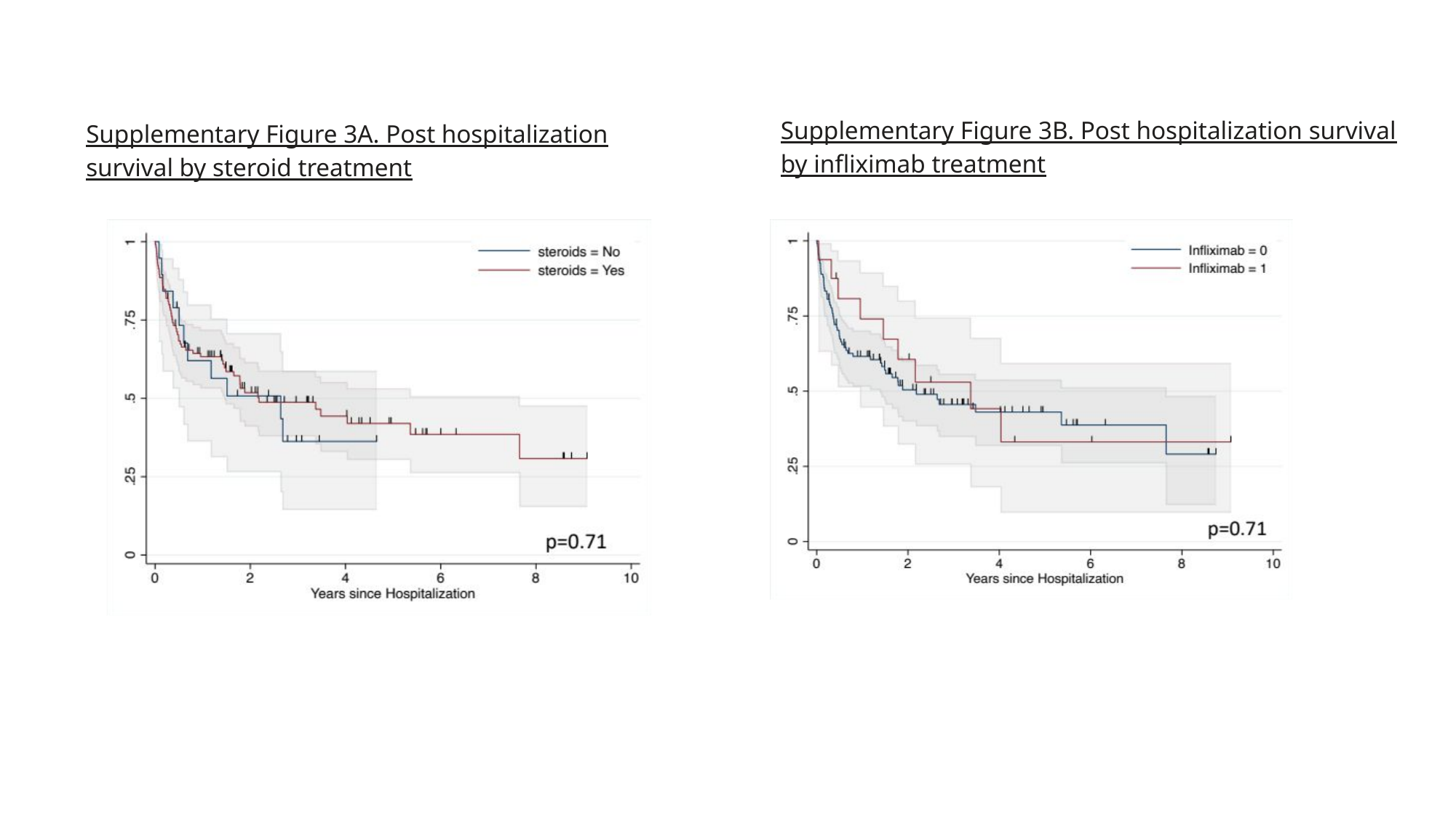

Supplementary Figure 3A. Post hospitalization survival by steroid treatment
Supplementary Figure 3B. Post hospitalization survival by infliximab treatment

## Slide 6
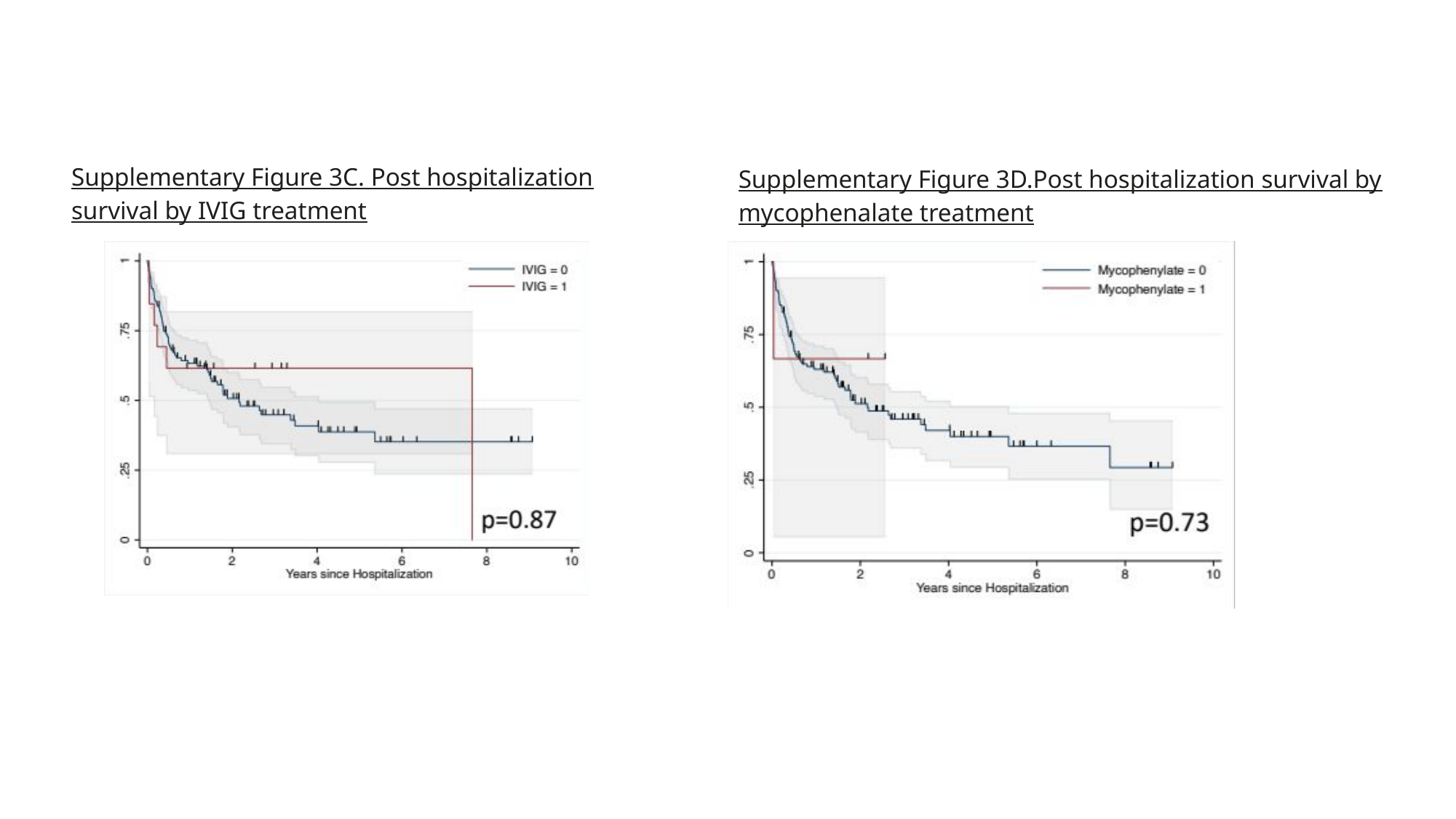

Supplementary Figure 3C. Post hospitalization survival by IVIG treatment
Supplementary Figure 3D.Post hospitalization survival by mycophenalate treatment

## Slide 7
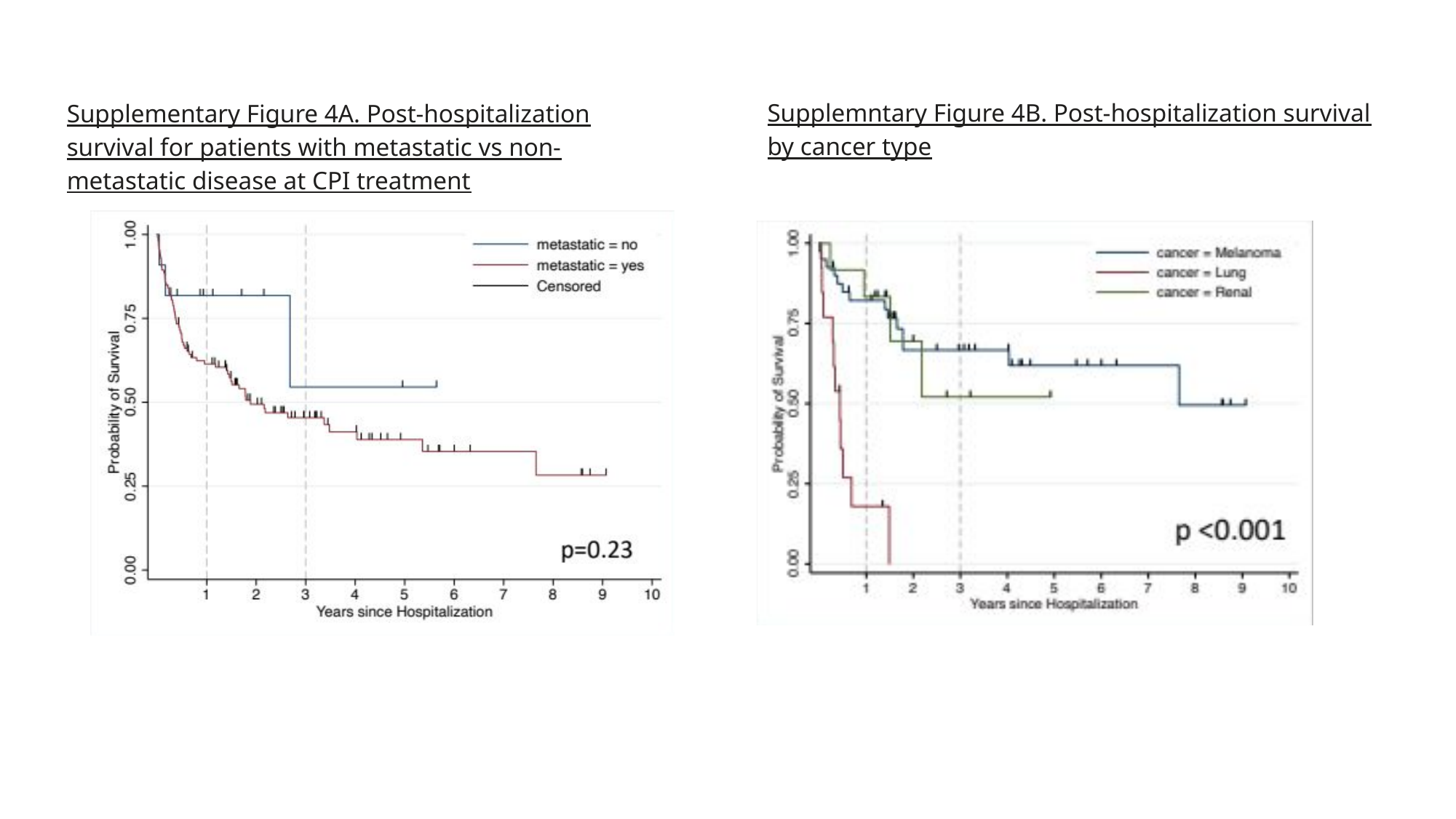

Supplemntary Figure 4B. Post-hospitalization survival by cancer type
Supplementary Figure 4A. Post-hospitalization survival for patients with metastatic vs non-metastatic disease at CPI treatment

## Slide 8
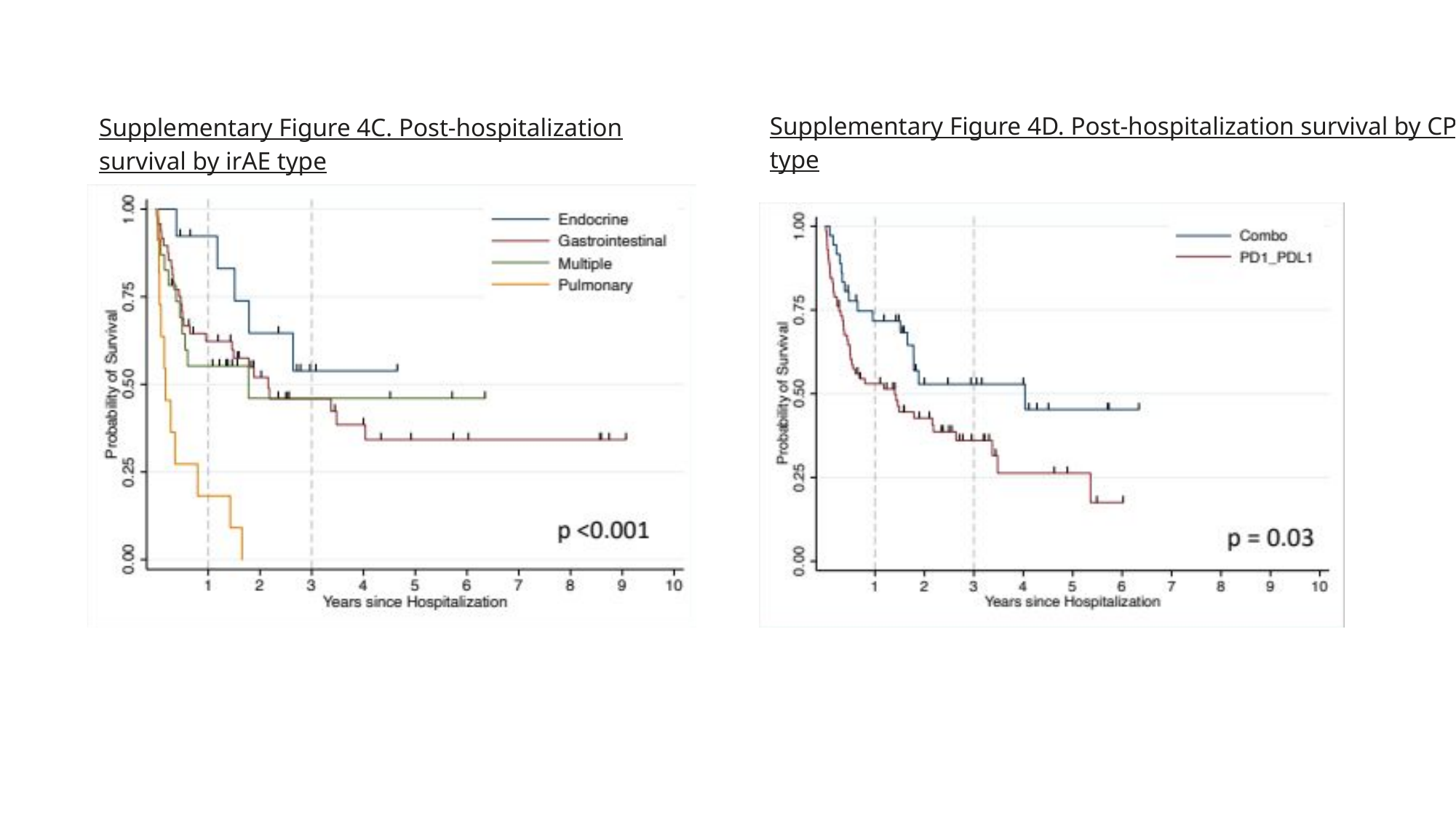

Supplementary Figure 4D. Post-hospitalization survival by CPI type
Supplementary Figure 4C. Post-hospitalization survival by irAE type
